# Dimensionality and invariance of ADL, IADL, BI-M2/WG-SS, and GALI in large surveys in France (2008–2014) and implications for measuring disability in epidemiology

**DOI:** 10.1186/s13690-023-01164-6

**Published:** 2023-08-07

**Authors:** Joël Coste, Karine Pérès, Jean-Marie Robine, Laure Carcaillon-Bentata

**Affiliations:** 1 Santé publique France (French national public health agency), Saint-Maurice, France; 2 University of Bordeaux, INSERM, Bordeaux Population Health, U1219 Bordeaux, France; 3 3MMDN, University of Montpellier, EPHE, INSERM, Montpellier, France; 4 PSL Research University, Paris, France

**Keywords:** Disability, Measurement, Rasch model, Differential item functioning, Unidimensionality, Incommensurability

## Abstract

**Background:**

The epidemiological investigation and surveillance of disability requires well-constructed, invariant, and, if possible, exchangeable measures. However, the current or recommended measures have not been thoroughly investigated with respect to these issues. Here we examined the dimensional structure and invariance of four measures across sociodemographic groups: Activities of Daily Living (ADL), Instrumental Activities of Daily Living (IADL), Budapest Initiative Mark 2 (BI-M2) and Washington Group on Disability Statistics Short Set (WG-SS), and Global Activity Limitation Indicator (GALI).

**Methods:**

We used data from three large nationwide representative surveys conducted in France between 2008 and 2014. The surveys included these four measures and classical and modern approaches (correlations, principal component analysis, Rasch modeling) were used to assess their dimensional structure as well as their invariance through differential item functioning (DIF) for sociodemographic characteristics. Polytomous logistic regression models were used to assess gradients in health inequalities associated with these measures.

**Results:**

For many items of ADL, IADL, and BI-M2/WG-SS, we consistently observed disordered response thresholds, rejection of unidimensionality, and DIF evidence for sociodemographic characteristics across the survey samples. Health inequality gradients were erratic. In addition, it was impossible to identify a common continuum for GALI, ADL, IADL, and BI-M2/WG-SS or their constituent items.

**Conclusion:**

This study warns against the current practice of investigating disability in epidemiology using measures that are unsuitable for epidemiological use, incommensurable, and inadequate regarding the basic requisites of dimensionality and invariance. Developing invariant measures and equating them along a common continuum to enlarge the common bases of measurement should therefore be a priority.

**Supplementary Information:**

The online version contains supplementary material available at 10.1186/s13690-023-01164-6.

**Table:** 

Text box 1. Contributions to the literature
• Epidemiological investigation and surveillance of disability require well-constructed, invariant, and, preferably, exchangeable measures
• The commonly used measures are not investigated regarding these issues
• We showed that Activities of Daily Living, Instrumental Activities of Daily Living, Budapest Initiative Mark 2, Washington Group on Disability Statistics Short Set, and Global Activity Limitation Indicator were incommensurable, with at least three of them being inadequate regarding dimensionality and invariance
• These findings caution against the current practice of using inappropriate measures for epidemiological research
• Invariant measures that can be equated along a common continuum should be developed to enlarge the common basis of disability measures

## Background

Disability is an important phenomenon that requires public health strategies as well as epidemiological investigation and surveillance. Several measures are commonly used in population surveys to assess disability, including single-item measures such as the Global Activity Limitation Indicator (GALI) [1], global questions used by the US National Center for Health Statistics [2], or sets of questions such as the Activities of Daily Living (ADL) [3] and Instrumental ADL (IADL) [4], which are also used to compute disability-free life expectancy [2, 5, 6]. More recently, items from the Budapest Initiative Mark (BI-M2) and Washington Group on Disability Statistics Short Set (WG-SS) have been proposed as disability measures “for use in censuses and surveys” [7]. ADL, IADL, and WG-SS are composite scales summing the responses to ordinal or Likert-type items to supposedly measure disability; they are used as ordinal or quantitative measures or scores. These measures are also widely used to compare heterogeneous populations across sociodemographic groups and to document health inequalities [8–11].

To date, no study has thoroughly examined, especially in a comparative and combined analysis, whether these measures can be used to assess a single unidimensional construct related to a common (single) continuum along which evaluated subjects can be ordered, and whether they are invariant or free of differential functioning (DIF: when external variables influence the endorsement of items and create biases in the measurement between subgroups defined by these variables) for the main demographic and socioeconomic characteristics. The properties of unidimensionality and invariance are of paramount importance with regard to the current epidemiological use of these measures. In addition, the measures that share the same continuum can be considered “exchangeable,” that is, they may be equated or co-calibrated using appropriate statistical techniques [12, 13].

Unidimensionality, DIF, and exchangeability of measures and scales can be investigated using modern measurement methods such as item response theory (IRT) and especially Rasch models. These models constitute a class of latent-trait models that are particularly appropriate for exploring the dimensionality of an item collection, determining their relative positions (referred to as “difficulties”) along the identified dimension, and identifying DIF items [14, 15]. It is especially important to identify such items with DIF, because they violate the requirement of unidimensionality, as the simple sum of the items is not a valid indicator of the underlying dimension. Rasch models are increasingly used to develop and refine composite health scales, especially to identify items that are redundant or poorly correlated with other items in a given dimension [16], to develop short versions of measurement instruments [17], and to evaluate the validity of instruments [18]. Rasch analysis also proves useful for addressing diagnostic problems such as the validity of diagnostic tests (with or without a reference standard) and assessing the influence of external covariates, which is generally not possible with classical methods such as logistic regression [19–21].

Over the past two decades, IRT and Rasch models have been applied to disability measurements (usually as complements to classical methods such as correlation and factor analysis) in numerous studies that aimed to: 1) investigate the psychometric properties of the newly developed WHO Disability Assessment Schedule (WHODAS) [22, 23] and the Model Disability Survey (MDS) instruments of the WHO and World Bank [24–26]; 2) investigate stability over time and settings of measures based on ADL, IADL, or both [27–31]; and 3) develop new measures of functioning using existing ADL and IADL subsets [32–35]. Though not the specific aims of these studies, various problems associated with ADL and IADL items have been identified concerning the response categories [27–29, 32, 35], strength of unidimensional continuum and redundancy of items [31–33], and DIF with regard to gender [30, 34, 35] and age [34–36]. The main objectives of our study were to evaluate the dimensional structure of ADL, IADL, WG-SS, and GALI measures and the extent to which they are affected by DIF regarding demographic and socioeconomic characteristics. Specifically, we aimed to respond to the following questions: 1) For the individual multi-item scales (ADL, IADL, BI-M2/WG-SS), are the scale items appropriately scored? Are the levels of responses (“difficulties”) relevant and appropriately distributed along the continuum? Are some items affected by DIF? How do they impact health inequality gradients? 2) For the scales as a whole, can these scales and their constitutive items be placed along the same continuum, thus allowing us to equate two or several scales?

## Methods

### Survey designs and study populations

We used data from two large nationwide representative surveys recently conducted in France using the same four measures of disability (GALI, ADL, IADL, and BI-M2/WG-SS).

First, the Disability Healthcare Household Survey (Enquête Handicap–Santé Ménages, HSM) is a cross-sectional two-stage survey that was conducted in 2008 with a focus on health, disability, and dependency. The participation rate was 80% for the first stage, which involved identifying individuals with potential disabilities. Note that this first stage did not involve screening strictly speaking but rather aimed to overrepresent subjects with disability in the study sample for the subsequent stage of the survey. For the second stage, the participation rate was 77%, leading to 23,348 participants aged ≥ 25 years living in France evaluated in face-to-face interviews and self-administered questionnaires [37].

Second, the Health, Healthcare, and Insurance Survey was conducted in two waves in 2012 and 2014 (Enquête Santé et Protection Sociale, ESPS). ESPS is a health survey representative of individuals living in households in France (95% of the total population), which collected information about their health status through telephone and face-to-face interviews conducted by specially trained interviewers as well as self-administered questionnaires [38, 39]. In 2012 and 2014, the participation rates were 66% and 64%, respectively, resulting in 15,315 and 17,593 participants aged ≥ 25 years.

Both HSM 2008 (the last available nationwide survey on disability in France) and ESPS 2012 and 2014 received institutional review board approval, and participants provided written informed consent.

### Measures of disability

The following measures of disability were recorded in the three surveys:

1) ADL. Six main activities: bathing, dressing, feeding, toileting, transferring (from bed, from chair), and walking [3];

2) IADL. Six main activities: shopping, doing light housework, doing heavy housework, preparing meals, handling finances, and using the telephone [4];

3) WG-SS. Six activities referred to as “basic activities” instead of “complex activities” [7]: seeing, hearing, walking or climbing steps, washing and dressing, remembering or concentrating, and communicating. BI-M2 includes the same activities as WG-SS with the exception of communicating (not assessed). In HSM, all WG-SS activities were recorded, whereas only BI-M2 activities were recorded in ESPS 2012 and 2014, with cognition assessed by remembering in 2012 and concentrating in 2014.

All these activities were rated on the same four-point response scale: no difficulty in performing the activity, some difficulty, much difficulty, and unable to do alone. The only exception was remembering, which was assessed in a binary format (no/yes).

4) The GALI question “For at least the past 6 months, to what extent have you been limited because of a health problem in activities people usually do?” was rated as “severely limited,” “limited but not severely,” and “not limited at all” following the recommendations [1].

To allow comparisons with GALI, trichotomous measures were further constructed for ADL and IADL using the five-class categorization of Stineman et al. [40], with groupings of “mild” and “moderate” as well as “severe” and “complete.” For BI-M2 and WG-SS, subjects who responded “much difficulty” or “unable to do alone” to any of the five or six questions were coded as “with severe disability”; participants who reported difficulty with any of the activities were separated from those who did not.

### Other variables

All three surveys recorded the following variables in the same way: age (years), gender (male, female), marital status (couple or single), education level (three categories: less than secondary, secondary, and tertiary), employment grade (four categories: manager or professional, middle manager or teacher, other employee or manual worker, no occupation or student), and income (in three terciles if provided, otherwise “not provided”).

### Statistical analysis

Statistical analysis was carried out in several steps. First, survey samples were described. Second, Spearman correlation matrices were constructed for disability measure items, followed by principal component analysis (based on polychoric correlations) with varimax and promax rotations of the components to be retained based on the most recommendable methods: Horn’s parallel analysis and Velicer’s minimum average partial test [41]. Third, a series of Rasch analyses were performed to address the specific questions posed by the multi-item scale measures (ADL, IADL, BI-M2/WG-SS): 1) difficulty thresholds for item responses and their order; 2) item fit and scale dimensionality and local independence; 3) uniform and non-uniform DIF and item bias for sociodemographic variables (0.1 logit was used as the threshold to indicate meaningful DIF [42]); 4) measurement precision and ability to discriminate between different levels of disability assessed by the person separation index (PSI). Fourth, another series of Rasch analyses was performed to address the possibility of defining a single continuum of disability on which disability measures, including GALI, can be located. The Rasch partial credit model, which is appropriate for ordered response categories, was used in the third and fourth steps. Fifth, associations between disability measures and sociodemographic variables were assessed using logistic regression models, odds ratios (OR), and 95% confidence intervals (CI).

As the largest and most comprehensive survey, HSM 2008 provided data for the main study, although replications with the other surveys were systematically performed due to the large samples (generating associations of limited value and possibly explained by oversampling).

SAS (version 9.4) and Rasch Unidimensional Measurement Modelling (RUMM) 2020 software were used for all the analyses, and appropriate weights were used to provide valid estimates for the French population (2008 for HSM, 2012 and 2014 for ESPS), while taking into account the unequal probabilities of selection resulting from sample design, non-responses, and non-coverage in both surveys [37–39].

## Results

### Subject characteristics, disability measure item characteristics, and correlations

The main characteristics of the three survey samples are presented in Supplementary Table 1. These samples were very similar in terms of gender, age, socioeconomic status, and disability measures, as both surveys were designed to reflect the French general population in 2008–2014. Limitations of activities associated with BI-M2/WG-SS, ADL, and IADL measures varied widely from about 1% (toileting, using the telephone) to 30% (GALI). For many ADL and especially IADL activities, intermediate categories of responses, especially “much difficulty,” were less frequently chosen than the extreme responses (“unable to do alone”).

Spearman correlation coefficients of the disability measures items are presented in Table 1 (19 items, HSM survey) and Supplementary Table 2 (18 items, ESPS 2012 and 2014 surveys). Excluding the trivial correlations between two WG-SS items (walking or climbing steps and washing and dressing) and three ADL items (bathing, dressing, and walking), Spearman coefficients ranged from 0.09 to 0.75 (median = 0.38), indicating only moderate correlations between the items overall (for the three surveys). The median of correlation coefficients was lower among BI-M2/WG-SS items (0.22 for the three surveys) than among ADL (0.55) or IADL items (0.48). The median correlation coefficient of GALI with the other items was also moderate (0.34), with the highest correlations observed with items relating to more physical activities. The principal component analysis performed for the 19 considered items brought to light two factors, with BI-M2/WG-SS items (with the exception of walking or climbing steps and washing and dressing) being clearly separated from both ADL and IADL items and GALI (Supplementary Table 3).

**Table 1:** Spearman's correlation coefficient matrix between individual items of WG-SS, ADL, IADL, and GALI (HSM survey)

	WF-SS items						ADL items						IADL items						GALI
	Seeing	Hearing	Washing and dressing	Walking or climbing steps	Remembering or concentrating	Communicating	Feeding	Toileting	Dressing	Bathing	Transferring from bed or chair	Walking	Shopping	Preparing meals	Doing light housework	Doing heavy housework	Handling finances	Using telephone	
Seeing	1.00	0.22	0.22	0.30	0.24	0.20	0.22	0.17	0.21	0.22	0.19	0.29	0.29	0.25	0.28	0.27	0.30	0.21	0.26
Hearing		1.00	0.18	0.24	0.26	0.24	0.13	0.09	0.15	0.17	0.12	0.22	0.20	0.16	0.19	0.21	0.20	0.13	0.23
Washing and dressing			1.00	0.52	0.26	0.30	0.57	0.50	0.86	0.92	0.59	0.55	0.59	0.61	0.62	0.57	0.49	0.41	0.41
Walking or climbing steps				1.00	0.29	0.23	0.35	0.30	0.46	0.49	0.40	0.87	0.62	0.45	0.58	0.61	0.46	0.24	0.61
Remembering or concentrating					1.00	0.37	0.20	0.17	0.23	0.26	0.20	0.26	0.29	0.27	0.29	0.30	0.34	0.23	0.33
Communicating						1.00	0.30	0.25	0.28	0.30	0.24	0.23	0.32	0.36	0.30	0.26	0.42	0.38	0.26
Feeding							1.00	0.55	0.60	0.56	0.50	0.38	0.44	0.57	0.48	0.42	0.42	0.47	0.28
Toileting								1.00	0.55	0.52	0.62	0.34	0.36	0.47	0.39	0.34	0.35	0.44	0.22
Dressing									1.00	0.73	0.63	0.48	0.51	0.58	0.55	0.50	0.43	0.41	0.36
Bathing										1.00	0.58	0.52	0.58	0.62	0.61	0.56	0.50	0.42	0.39
Transferring from bed or chair											1.00	0.43	0.44	0.48	0.47	0.43	0.37	0.35	0.29
Walking												1.00	0.64	0.48	0.59	0.61	0.48	0.26	0.56
Shopping													1.00	0.65	0.71	0.72	0.65	0.39	0.50
Preparing meals														1.00	0.68	0.58	0.61	0.51	0.38
Doing light housework															1.00	0.75	0.58	0.38	0.48
Doing heavy housework																1.00	0.57	0.34	0.50
Handling finances																	1.00	0.46	0.39
Using telephone																		1.00	0.23
GALI																			1.00

### Rasch analyses of separate BI-M2/WG-SS, ADL, and IADL measures

The first Rasch analyses performed using the original four-category coding of BI-M2/WG-SS, ADL, and IADL items revealed almost generalized disordered thresholds (Supplementary Fig. 1), which persisted after recoding the items into three categories (grouping “much difficulty” with “unable to do alone”; Supplementary Fig. 2). This phenomenon of disordered thresholds was consistently observed across surveys (ESPS surveys: data not shown). Further analyses were then performed using binary items (“any limitation” vs “not limited” for the given activity).

**Fig. 1:**
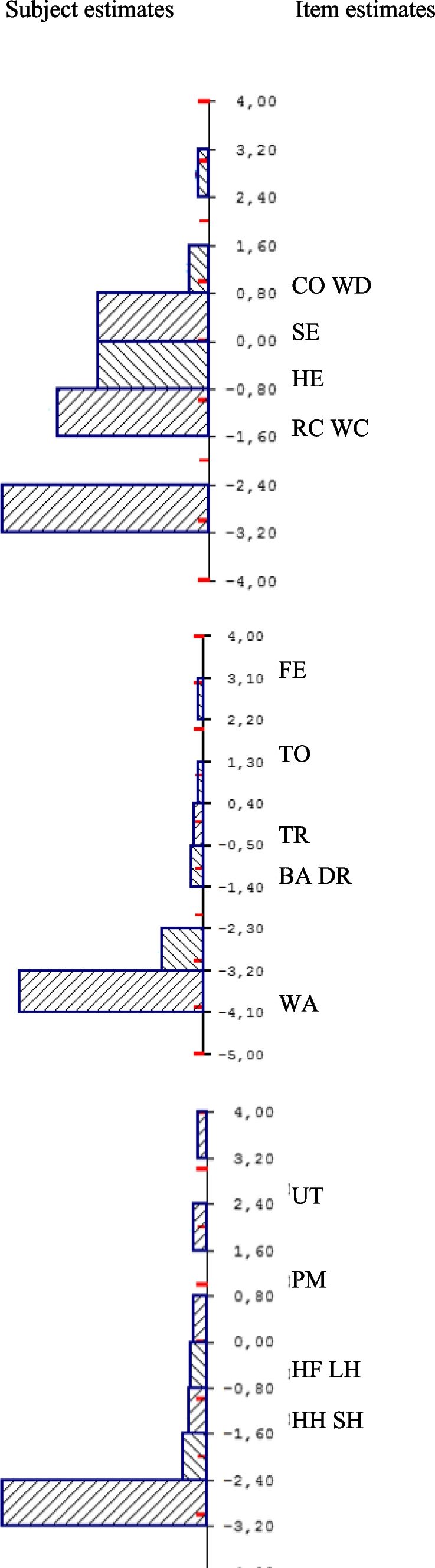
Subject-item maps of the WG-SS, ADL, and IADL items (two-category responses or one threshold: “some difficulty or more”). HSM survey. On the left of the diagram are the subjects, and on the right are the thresholds of each item (point on the continuum where the response category “some difficulty or more” is most likely to be chosen by a subject with the corresponding level of disability). Less disabled subjects are near the bottom of the diagram, and most disabled subjects are near the top. Abbreviations SE: Seeing, HE: Hearing, WD: Washing and dressing, WC: Walking or climbing steps, RC: Remembering or concentrating, CO: Communicating. FE: Feeding, TO: Toileting, DR: Dressing, BA: Bathing, TR: Transferring from bed or chair, WA: Walking. SH: Shopping, PM: Preparing meals, LH: Doing light housework, HH: Doing heavy housework, HF: Handling finances, UT: Using telephone

Table 2 and Supplementary Tables 4 and 5 present summaries of the Rasch analyses of BI-M2/WG-SS, ADL, and IADL items (recoded as two categories). A strong misfit to the unidimensional Rasch model was found for ADL and IADL items in the three surveys (total-item chi-square test for the item-trait interaction *p* < 10^–5^) and in one survey (ESPS 2012) for WG-SS/BI-M2 items. WG-SS/BI-M2 was especially characterized by a low person separation index (0.34 to 0.60 depending on the survey). The subject-item maps shown in Fig. 1 provide evidence that the items are not well distributed in most measures, especially WG-SS/BI-M2 for which many items are located in the middle part of the disability spectrum. No local dependence was found in any sets of items.

**Table 2:** Rasch analyses of dimensionality and differential item functioning (DIF^a^) for WG-SS, ADL, and IADL items (recoded as binary variables, limited vs non-limited) (HSM survey)

**WG-SS items**	Fit with the one-dimensional Rasch model			Differential Item Functioning	
Overall fit *p*-value = 0.88, Person separation index = 0.60	location	Chi square	*P*-value	Uniform DIF	Non-Uniform DIF
Seeing	0.30	1.16	0.88	–	–
Hearing	-0.21	0.69	0.95	–	–
Washing and dressing	1.04	5.53	0.24	age	–
Walking or climbing steps	-0.88	4.57	0.33	age	–
Remembering or concentrating	-1.27	0.53	0.97	–	–
Communicating	1.01	3.53	0.47	gender, age, occupation	–
**ADL items**	Fit with the one-dimensional Rasch model			Differential Item Functioning	
Overall fit *p*-value < 0.0000001, Person separation index = 0.83	location	Chi square	*P*-value	Uniform DIF	Non-Uniform DIF
Feeding	3.56	2.61	0.46	–	–
Toileting	0.05	7.20	0.07	–	–
Dressing	-0.98	18.34	0.0004	–	–
Bathing	2.09	2.86	0.41	–	–
Transferring from bed or chair	-1.26	23.55	0.00003	–	–
Walking	-3.45	28.70	0.000003	–	–
**IADL items**	Fit with the one-dimensional Rasch model			Differential Item Functioning	
Overall fit *p*-value = 0.0003, Person separation index = 0.87	location	Chi square	*P*-value	Uniform DIF	Non-Uniform DIF
Shopping	-1.17	10.53	0.03	–	–
Preparing meals	0.96	14.82	0.01	gender	–
Doing light housework	-0.67	9.91	0.04	gender	–
Doing heavy housework	-1.32	2.03	0.73	–	–
Handling finances	-0.51	9.60	0.05	education	–
Using telephone	2.71	8.76	0.07	–	–

^a^A difference of 0.1 logit was considered to indicate meaningful DIF (see text)

Whereas all ADL items were free of DIF, five/six BI-M2/WG-SS items and five/six IADL items were affected by meaningful DIF in at least one analysis (Table 2, Supplementary Tables 4 and 5). Gender was associated with uniform DIF in six items, occupation in five, age in four, coupled status and education in three, and income in one. Gender-associated DIF was observed in both directions with several items such as limitations in light or heavy housework and shopping being endorsed at lower levels by women and limitations in preparing meals at lower levels by men. By contrast, DIF associated with other sociodemographic variables consistently concerned older, less educated, less skilled, and subjects living alone who endorsed the limitations at lower levels.

### Rasch analyses of composite measures

Severely disordered thresholds were also observed for the five-category ADL and IADL measures (Supplementary Fig. 3), which required further Rasch analyses using dichotomized measures (“any limitation” vs “not limited”). However, it was not possible to situate ADL, IADL, WG-SS, and GALI or even ADL, IADL, and GALI on the same unidimensional continuum (Table 3). In these analyses, WG-SS was endorsed at lower levels of disability compared to GALI, IADL, and especially ADL. In this context, DIF analysis is challenging due to the rejection of unidimensionality, although WG-SS and IADL were found to be severely affected by age, education, and occupation DIF in one sample, whereas GALI did not seem completely free of DIF with regard to age and education in another sample. When we attempted to construct a set of ADL and IADL items free of DIF (Table 4), the result was neither totally satisfactory (due to persisting gender DIF) nor reproducible across surveys. Restricting the analyses to subjects over 65 years did not change the results (data not shown).

**Table 3:** Rasch analyses of dimensionality and differential item functioning (DIF) for the overall WG-SS, ADL, IADL, and GALI indicators (recoded as binary variables, limited vs non-limited) (HSM survey)

**HSM Survey**	Fit with the one-dimensional Rasch model			Differential Item Functioning	
Overall fit *p*-value = 0.006, Person separation index = 0.79	location	Chi square	*P*-value	Uniform DIF	Non-Uniform DIF
WG-SS	-2.03	4.21	0.12	–	–
ADL	1.86	8.83	0.01	–	–
IADL	1.42	3.32	0.19	–	–
GALI	-1.26	5.20	0.07	–	–
**ESPS 2012 Survey**	Fit with the one-dimensional Rasch model			Differential Item Functioning	
Overall fit *p*-value = 0.00006, Person separation index = 0.68	location	Chi square	*P*-value	Uniform DIF	Non-Uniform DIF
WG-SS	-1.79	8.92	0.01	age, education, occupation	–
ADL	2.13	3.99	0.14	–	–
IADL	0.03	7.86	0.02	age, education, occupation	–
GALI	-0.37	12.36	0.002	–	–
**ESPS 2014 Survey**	Fit with the one-dimensional Rasch model			Differential Item Functioning	
Overall fit *p*-value = 0.00001, Person separation index = 0.72	location	Chi square	*P*-value	Uniform DIF	Non-Uniform DIF
WG-SS	-2.16	11.06	0.004	–	–
ADL	2.59	3.00	0.22	–	–
IADL	0.12	10.12	0.01	–	–
GALI	-0.55	12.76	0.002	age	–
Rasch analyses of dimensionality and differential item functioning (DIF) for the overall WG-SS, ADL, IADL, and GALI indicators (recoded as binary variables, limited vs non-limited)					
**HSM Survey**	Fit with the one-dimensional Rasch model			Differential Item Functioning	
Overall fit *p*-value < 0.000001, Person separation index = 0.75	location	Chi square	*P*-value	Uniform DIF	Non-Uniform DIF
ADL	1.08	14.18	0.0001	–	–
IADL	0.76	11.52	0.001	–	–
GALI	-1.84	13.95	0.0002	–	–
**ESPS 2012 Survey**	Fit with the one-dimensional Rasch model			Differential Item Functioning	
Overall fit *p*-value = 0.007, Person separation index = 0.69	location	Chi square	*P*-value	Uniform DIF	Non-Uniform DIF
ADL	1.43	96.19	< 0.000001	–	–
IADL	-0.50	91.83	< 0.000001	–	–
GALI	-0.93	47.48	< 0.000001	–	–
**ESPS 2014 Survey**	Fit with the one-dimensional Rasch model			Differential Item Functioning	
Overall fit *p*-value = 0.0001, Person separation index = 0.72	location	Chi square	*P*-value	Uniform DIF	Non-Uniform DIF
ADL	1.77	6.52	0.010	–	–
IADL	-0.55	8.73	0.003	–	–
GALI	-1.22	5.06	0.020	age, education	–

**Table 4:** Rasch analyses of dimensionality and differential item functioning (DIF) for a selected set of ADL and IADL items (HSM survey). The best selection in terms of fit and DIF is presented

**HSM Survey**	Fit with the one-dimensional Rasch model			Differential Item Functioning	
Overall fit *p*-value = 0.03, Person separation index = 0.88	location	Chi square	*P*-value	Uniform DIF	Non-Uniform DIF
Feeding	3.79	0.49	0.99	–	–
Toileting	2.42	4.21	0.52	–	–
Dressing	-0.26	5.65	0.34	–	–
Bathing	-0.49	9.43	0.09	–	–
Transferring from bed or chair	0.63	1.86	0.87	–	–
Walking	-2.79	15.87	0.01	–	–
Shopping	-2.31	12.78	0.03	gender	–
Preparing meals	-0.43	7.64	0.18	–	–
Doing light housework	-1.92	8.43	0.13	–	–
Using telephone	1.36	5.83	0.32	–	–
**ESPS 2012 Survey**	Fit with the one-dimensional Rasch model			Differential Item Functioning	
Overall fit *p*-value = 0.03, Person separation index = 0.87	location	Chi square	*P*-value	Uniform DIF	Non-Uniform DIF
Feeding	1.90	1.98	0.85	–	–
Toileting	1.67	6.62	0.25	–	–
Dressing	0.01	7.35	0.20	–	–
Bathing	0.30	12.85	0.02	–	–
Transferring from bed or chair	-0.16	4.24	0.52	–	–
Walking	-1.49	4.58	0.47	gender	–
Shopping	-1.25	12.40	0.03	gender	–
Preparing meals	-0.79	3.10	0.69	gender	–
Doing light housework	-1.19	11.43	0.04	–	–
Using telephone	1.00	5.55	0.35	–	–
Rasch analyses of dimensionality and differential item functioning (DIF) for a selected set of ADL and IADL items. The best selection in terms of fit and DIF is presented					
**ESPS 2014 Survey**	Fit with the one-dimensional Rasch model			Differential Item Functioning	
Overall fit *p*-value = 0.02, Person separation index = 0.88	location	Chi square	*P*-value	Uniform DIF	Non-Uniform DIF
Feeding	2.26	1.10	0.95	–	–
Toileting	2.01	8.26	0.14	–	–
Dressing	-0.05	8.97	0.11	–	–
Bathing	-0.13	13.53	0.02	–	–
Transferring from bed or chair	0.05	3.61	0.61	–	–
Walking	-2.09	1.95	0.86	–	–
Preparing meals	-0.90	3.95	0.56	gender	–
Doing light housework	-1.83	11.48	0.04	–	–
Using telephone	0.69	13.90	0.02	–	–

### Associations of disability measures with age, gender, and education level

Table 5 presents the associations of the disability measures recoded into two categories with sex, age, and education level in the HSM survey. Crude and adjusted ORs, obtained from logistic regression models, varied notably across measures. ORs of disability associated with the female gender were much greater with IADL, which is most affected by gender DIF. On the contrary, ORs associated with a lower education level are lower with GALI, which is free of DIF with regard to this variable in the HSM sample. Regarding age, comparisons are more difficult to interpret, since ADL and IADL were designed for use in the elderly (see the Discussion below).

**Table 5:** Associations of two-level disability (limited vs non-limited) indicators with sex, age, and education level (HSM survey). Odds ratios and 95% confidence intervals obtained in logistic regression models including one (crude) or all (adjusted) of these three determinants

	WG-SS		ADL		IADL		GALI	
	Crude	Adjusted	Crude	Adjusted	Crude	Adjusted	Crude	Adjusted
Sex, female	1.24 (1.15–1.35)	1.15 (1.05–1.26)	1.53 (1.40–1.69)	1.25 (1.13–1.39)	2.09 (1.90–2.30)	1.86 (1.67–2.07)	1.28 (1.18–1.38)	1.15 (1.05–1.25)
*Age*								
25–34 years	Ref	Ref	Ref	Ref	Ref	Ref	Ref	Ref
35–44 years	1.60 (1.35–1.90)	1.49 (1.26–1.78)	1.92 (1.32–2.78)	1.71 (1.18–2.48)	1.14 (0.85–1.53)	1.02 (0.76–1.37)	1.40 (1.15–1.69)	1.30 (1.07–1.58)
45–54 years	2.92 (2.47–3.44)	2.54 (2.15–3.01)	3.59 (2.55–5.05)	2.87 (2.04–4.05)	1.73 (1.31–2.29)	1.35 (1.02–1.79)	2.38 (1.99–2.87)	2.03 (1.73–2.51)
55–64 years	4.06 (3.44–4.79)	3.31 (2.80–3.93)	6.15 (4.39–8.61)	4.41 (3.14–6.18)	2.48 (1.88–3.27)	1.71 (1.28–2.29)	3.34 (2.79–4.00)	2.71 (2.25–3.26)
65–74 years	7.11 (5.94–8.52)	5.22 (4.33–6.29)	10.17 (7.25–14.26)	6.21 (4.42–8.73)	4.49 (3.40–5.93)	2.55 (1.89–3.44)	5.21 (4.31–6.30)	3.79 (3.12–4.61)
75–84 years	15.76 (12.81–19.40)	10.75 (8.67–13.34)	33.74 (24.14–47.15)	18.88 (13.45–26.49)	17.27 (13.13–22.73)	9.04 (6.74–12.12)	11.58 (9.51–14.10)	7.84 (6.39–9.62)
≥ 85 years	48.98 (31.73–75.61)	33.28 (21.48–51.57)	126.98 (87.12–185.08)	72.77 (49.84–106.25)	59.16 (42.62–82.13)	31.91 (22.59–45.07)	30.92 (22.89–41.75)	20.89 (15.42–28.28)
*Education*								
Less than secondary	4.83 (4.27–5.46)	2.46 (2.15–2.81)	9.96 (8.21–12.08)	3.82 (3.10–4.70)	9.35 (7.78–11.24)	4.23 (3.44–5.21)	4.55 (4.01–5.17)	2.36 (2.07–2.71)
Secondary	1.96 (1.74–2.20)	1.65 (1.46–1.86)	2.63 (2.15–3.21)	2.04 (1.65–2.53)	2.39 (1.97–2.89)	2.03 (1.66–2.47)	1.81 (1.59–2.06)	1.53 (1.34–1.74)
Tertiary	Ref	Ref	Ref	Ref	Ref	Ref	Ref	Ref

## Discussion

By adopting classical and especially modern measurement approaches, this study of four widely used or recommended measures of disability (GALI, ADL, IADL, and BI-M2/WG-SS) in three large representative general population samples conducted in France provided important insights into the functioning of these measures. Given the consistent observation of disordered response thresholds, the rejection of unidimensionality, and the evidence for DIF according to sociodemographic characteristics for many items (with consequences on the measures of association between disability and these sociodemographic characteristics), this study calls into question the use of most of these measures for disability surveillance in the general population. Moreover, the failure to identify a common continuum on which these measures or their constituent items can be placed excludes the formal comparison of data collected with one or another measure.

### Disordered thresholds

With the exception of GALI, all the studied measures repeatedly presented problems of disordered thresholds when using the commonly used four-category and even the regrouped three-category responses of these measures. The disordering of thresholds, a phenomenon evidenced by partial credit Rasch models, usually indicates that too many categories are offered to and chosen by respondents. This issue is usually resolved by collapsing the categories with reverted thresholds. However, this may also indicate multidimensionality, especially when middle categories measure something different from the concept associated with the unidimensional continuum [43]. Regarding ADL and IADL, disordered thresholds have already been reported in several studies [27–29, 32, 35], although the implications, which require more than just collapsing categories, have not yet been dealt with. Regarding BI-M2/WG-SS, the reference to composite activities (walking *or* climbing steps, washing all over *and* dressing, remembering *or* concentrating) in three items is probably non-optimal in this respect. Of note, GALI and the trichotomous measure of ADL derived from the categorization of Stineman et al. [40] were free of disordered thresholds in our surveys.

### Dimensionality problems

The strong rejection of the hypotheses of unidimensional continuums for ADL, IADL, and BI-M2/WG-SS in most of the analyses performed in this study are especially problematic. The summation of responses to a set of items implies that all the items measure the same underlying trait – here, disability –, which allows subjects to be positioned along a continuum from “very able” to “very disabled.” If this property of unidimensional continuum is not met, then two or more traits are entangled, and inferences based on the summated score are not valid and cannot be used with confidence. In the case of ADL and IADL items, these problems have already been reported [32], although most publications presenting Rasch analyses *simultaneously* consider ADL and IADL activities along the same continuum [27–29, 31, 35]: this approach probably minimizes the dimensionality problems after discarding the severely misfitting items, as observed in our study. Indeed, excluding 2–3 items allowed us to avoid the rejection of unidimensionality in the three surveys, although the final set of items was not the same.

### Differential item functioning

As reported in previous Rasch studies on disability [30, 34–36], we found that many IADL and BI-M2/WG-SS items were plagued by meaningful DIF (> 0.1 logit) for age and especially gender, as well as occupation, education, and marital status, with obvious consequences when assessing the association between disability and sociodemographic determinants. Precisely quantifying health inequalities in terms of disability in order to reduce them requires the use of invariant measures across these major determinants and not rubber bands [44]. In particular, the use of “gendered” activities, although relevant in clinical settings, should be carefully considered in epidemiological contexts, especially when stratification is unusual. On the contrary, no or limited DIF problems were observed with ADL and GALI, although their constitutive items were tested on uncertain continuums in this study, indicating that our results are not completely reliable. In particular, GALI, which has already been used to investigate health inequalities in different countries [45], merits further examination, particularly with regard to its invariance across the main the sociodemographic determinants.

### Lack of a single continuum for disability measures

Despite testing various combinations of ADL and IADL items with BI-M2/WG-SS items and GALI, it was not possible to construct a single continuum that includes these measures or even a subset of their constituting items, although the endeavor was almost successful for ADL and IADL (see above). GALI and BI-M2/WG-SS items relating to functional limitations (i.e., seeing, hearing, remembering, or concentrating) consistently diverged from ADL and IADL activities, meaning that it was impossible to calibrate or equate these instruments. To date, only physical functioning scales of multidimensional health-related quality of life have been successfully equated [46–48]. As a result, disability-free life expectancy, computed in many countries using ADL (sometimes IADL) or GALI [49], should be considered incommensurable.

### Study strengths and limitations

This study has several strengths: 1) the use of three large general population samples instead of convenience clinical samples most often used in other studies; 2) the concomitant use of several commonly used measures of disability; and 3) the replicated analyses and consistent results obtained across samples and measures, which provide robust evidence in response to the research questions. The study has also several limitations: 1) its purely cross-sectional design and unrepeated measures, thus preventing the assessment of their reliability (which might have informed strategies to select a subset of items); 2) the absence of any external assessment (e.g., clinical or by proxy) of disability; and 3) possible (unmeasured) confounding, notably due to cognitive performances, which was not considered per se in this study.

### Implications for measuring disability in populations: Use of existing instruments

Several findings of this study have implications on the current practice of measuring disability for epidemiological investigation and surveillance. We showed that popular instruments such as ADL and IADL, which are used to compute disability-free life expectancy in many countries, or the more recent BI-M2/WG-SS are plagued with dimensionality problems and widespread DIF (gender, age, as well as socioeconomic variables) that are likely to bias comparisons and estimations of inequalities. These problems, which have already been noted in previous studies, should be seriously considered and addressed. The incommensurability of ADL, IADL, BI-M2/WG-SS, and GALI identified in this study is another serious problem but not surprising, since these measures were initially developed for different purposes and not necessarily congruent with epidemiological use. First, the Katz ADL [3] aims to measure caregiver burden in nursing facilities, with the targeted activities being personal, basic, and essential for survival. Second, the Lawton IADL [4] targets activities necessary to live independently in the community; these activities are household rather than personal tasks. ADL and IADL are widely used in the clinical and social fields of geriatrics. Third, GALI was developed in Europe within the framework of the disability concepts of Nagi, Wood, and Verbrugge to allow for comparisons of usual activities (or their limitations) across populations in time and space [1]. ADL, IADL, and GALI therefore share the same target in terms of activities and limitations of activities. Fourth, the BI-M2/WG-SS instruments aggregate items related to functional limitations (i.e., seeing, hearing, remembering, concentrating), daily activities (i.e., walking, climbing steps, washing, dressing), and communication. Despite aiming to “improve disability statistics” [50], these instruments have encountered various difficulties in their application [51, 52] and have not yet reached the popularity of previous measures. To these measures, we may add the WHODAS instruments (first version in 1988, second in 2010), with the most recent version being developed in the framework of the International Classification of Functions, with 36 questions addressing functions, activities, and participation [53]. Nevertheless, WHODAS is still not commonly used in population settings. Very recently, a short version of the Model Disability Survey (MDS) instrument of the WHO and World Bank was developed for use in epidemiology and surveys [24, 25]. Unlike the ADL, IADL, BI-M2/WG-SS, and GALI, this instrument benefitted from the use of modern measurement approaches (Rasch models) during its development. It also underwent formal psychometric evaluation with encouraging results from Afghanistan, Cameroon, Chile, Costa Rica, India, Laos, Pakistan, Philippines, Sri Lanka, and Tajikistan [26].

### Implications for measuring disability in populations: Future research

Our results, which bring to light conceptual divergences, also support the urgent need to dismantle the ever-growing “Tower of Babel” of disability measures and instead develop sample-free and scale-free measurements. Instead of endlessly developing new instruments, infinitely varying the combinations of activities or levels of responses [54], or mixing activities with different types of functioning and participation, which is perhaps even worse, researchers, with the encouragement of decision-makers, should rather focus on developing invariant measures and equating them in order to identify and enlarge the common bases of measurement with the aim to better study and monitor disability [12] and exploit from a comparative perspective the massive body of existing data. The example of the field of health-related quality of life, in which instruments or subscales (including physical functioning) have been equated for at least 25 years [46], should be taken as a source of inspiration. Theoretical reflections and empirical research on response modalities or “thresholds” of disability, which was once actively pursued (e.g., by Isaacs and Neville [55]), also probably deserve to be reinitiated. Calibrated item banks and computer adaptive testing approaches such as those developed for mental health or quality of life measurements may be desirable goals in the field of disability. The GALI, several DIF-free ADL and IADL items, and questions from the more recent WHODAS and MDS instruments may be considered as a priority for future developments.

## Conclusions

This study found that several measures commonly used to assess disability in populations are incommensurable and inadequate regarding the basic requirements of dimensionality and invariance. The current priority should therefore be to develop psychometrically sound and invariant measures for epidemiologic purposes and to equate them along a common continuum in order to enlarge the common bases of measurement.

### Supplementary Information

**Additional file 1: Supplementary Table 1.** Description of the studied samples (HSM and ESPS surveys). **Supplementary Table 2.** Spearman’s correlation coefficient matrix between individual items of BI-M2, ADL, IADL, and GALI. ESPS surveys, 2012 and 2014. **Supplementary Table 3.** Factor pattern matrices obtained using principal component analysis with varimax and promax rotations for the 19 items of the WG-SS, ADL, IADL, and GALI disability indicators. HSM survey. **Supplementary Table 4.** Rasch analyses of dimensionality and differential item functioning for the BI-M2, ADL, and IADL items (recoded as binary variables, limited vs non-limited). ESPS 2012 survey. **Supplementary Table 5.** Rasch analyses of dimensionality and differential item functioning for the BI-M2, ADL and IADL items (recoded as binary variables, limited vs non-limited). ESPS 2014 Survey. **Supplementary Fig. 1.** Subject-item maps of the WG-SS, ADL, and IADL items (four-category responses or three thresholds, 1: some difficulty, 2: much difficulty, 3: unable to do alone; two-category responses and one threshold: “some difficulty or more”). HSM survey. On the left of the diagram are the subjects, and on the right are the thresholds of each item (point on the continuum where the response category “some difficulty or more” is most likely to be chosen by a subject with the corresponding level of disability). Less disabled subjects are near the bottom of the diagram, and most disabled subjects are near the top. Abbreviations SE: Seeing, HE: Hearing, WD: Washing and dressing, WC: Walking or climbing steps, RC: Remembering or concentrating, CO: Communicating. FE: Feeding, TO: Toileting, DR: Dressing, BA: Bathing, TR: Transferring from bed or chair, WA: Walking. SH: Shopping, PM: Preparing meals, LH: Doing light housework, HH: Doing heavy housework, HF: Handling finances, UT: Using telephone. **Supplementary Fig. 2.** Subject-item maps of the WG-SS, ADL, and IADL items (three-category responses or two thresholds, 1: some difficulty, 2: much difficulty or unable to do alone). HSM survey. On the left of the diagram are the subjects, and on the right are the thresholds of each item (point on the continuum where the response category “some difficulty or more” is most likely to be chosen by a subject with the corresponding level of disability). Less disabled subjects are near the bottom of the diagram, and most disabled subjects are near the top. Abbreviations SE: Seeing, HE: Hearing, WD: Washing and dressing, WC: Walking or climbing steps, RC: Remembering or concentrating, CO: Communicating. FE: Feeding, TO: Toileting, DR: Dressing, BA: Bathing, TR: Transferring from bed or chair, WA: Walking. SH: Shopping, PM: Preparing meals, LH: Doing light housework, HH: Doing heavy housework, HF: Handling finances, UT: Using telephone. **Supplementary Fig. 3.** Subject-item maps of the ADL, IADL, WG-SS, and GALI items (three-category responses or two thresholds for WG-SS and GALI: 1: some difficulty, 2: much difficulty or unable to do alone; five-category responses or four thresholds for ADL and IADL according to Stineman et al. [37]). HSM survey. On the left of the diagram are the subjects, and on the right are the thresholds of each item (point on the continuum where the response category “some difficulty or more” is most likely to be chosen by a subject with the corresponding level of disability). Less disabled subjects are near the bottom of the diagram, and most disabled subjects are near the top. Abbreviations AD: ADL, IA: IADL, GA: GALI; WG: WGSS.

## Data Availability

The data that support the findings of this study are available from the Directorate for Research, Studies, Evaluation, and Statistics (DREES), the French National Institute of Statistics and Economic Studies (INSEE), and the French Institute for Research and Information in Health Economics (IRDES), although legal restrictions apply to the availability of these data, which were used under license for the current study and for which the authors do not have permission to make them publicly available. Interested researchers can request permission to access these data by contacting rf.vuog.etnas@SOFNI-SEERD, https://www.insee.fr/fr/information/2416123 (HSM 2008 survey), or rf.sedri@tcatnoc (ESPS 2010 survey).

## Abbreviations

ADL: Activities of Daily Living
BI-M2: Budapest Initiative Mark-2
CI: Confidence interval
DIF: Differential item functioning
ESPS: Enquête Santé et Protection Sociale
GALI: Global Activity Limitation Indicator
HSM: Enquête Handicap–Santé Ménages
IADL: Instrumental Activities of Daily Living
IRT: Item response theory
OR: Odds ratio
PSI: Person separation index
WG-SS: Washington Group on Disability Statistics Short Set
WHODAS: WHO Disability Assessment Schedule
