# Supplementary material for: Dimensionality and invariance of ADL, IADL, BI-M2/WG-SS, and GALI in large surveys in France (2008–2014) and implications for measuring disability in epidemiology
Source: Arch Public Health. 2023 Aug 7;81:141. doi: 10.1186/s13690-023-01164-6 (PMC10405560; doi:10.1186/s13690-023-01164-6)

Supplementary material

Supplementary Table 1. Description of the studied samples (HSM and ESPS surveys).

Supplementary Table 2. Spearman's correlation coefficient matrix between individual items of BI-M2, ADL, IADL, and GALI. ESPS surveys, 2012 and 2014.

Supplementary Table 3. Factor pattern matrices obtained using principal component analysis with varimax and promax rotations for the 19 items of the WG-SS, ADL, IADL, and GALI disability indicators. HSM survey.

Supplementary Table 4. Rasch analyses of dimensionality and differential item functioning for the BI-M2, ADL, and IADL items (recoded as binary variables, limited vs non-limited). ESPS 2012 survey.

Supplementary Table 5. Rasch analyses of dimensionality and differential item functioning for the BI-M2, ADL and IADL items (recoded as binary variables, limited vs non-limited). ESPS 2014 Survey.

Supplementary Fig. 1. Subject-item maps of the WG-SS, ADL, and IADL items (four-category responses or three thresholds, 1: some difficulty, 2: much difficulty, 3: unable to do alone; two-category responses and one threshold: "some difficulty or more"). HSM survey.

Supplementary Fig. 2. Subject-item maps of the WG-SS, ADL, and IADL items (three-category responses or two thresholds, 1: some difficulty, 2: much difficulty or unable to do alone). HSM survey.

Supplementary Fig. 3. Subject-item maps of the ADL, IADL, WG-SS, and GALI items (three-category responses or two thresholds for WG-SS and GALI: 1: some difficulty, 2: much difficulty or unable to do alone; five-category responses or four thresholds for ADL and IADL according to Stineman et al. [37]). HSM survey.

Supplementary Table 1 Description of the studied samples (HSM and ESPS surveys). All figures are weighted percentages unless otherwise indicated.

	HSM Survey	2012 ESPS Survey	2014 ESPS Survey
	(N=23,348)	(N=15,315)	(N=17,593)
Sex, female	52.8	53.5896	52.9213
Age			
25-34 years	17.8	17.4	17.6
35-44 years	21.3	19.3	18.8
45-54 years	20.3	18.5	19.5
55-64 years	17.7	18.8	18.2
65-74 years	11.8	12.3	13.5
75-84 years	8.6	9.7	8.8
≥ 85 years	2.6	4.1	3.6
Education			
Less than secondary	30.7	31.8	31.3
Secondary	44.6	40.7	37.2
Tertiary	24.7	27.5	31.5
Marital status			
Married/living with a partner	71.7	70.5	70.9
Separated/divorced/widowed	14.0	17.0	14.9
Single	14.3	12.5	14.2
Occupation (present or past)			
Manager, professional	15.4	17.1	18.8
Middle manager, teacher	41.6	38.6	41.5
Other, manual worker	41.8	41.2	35.5
No occupation or student	1.2	3.1	4.2
Household incomes			
Lower third	29.8	23.1	23.2
Middle third	32.3	33.5	35.3
Upper third	31.2	19.1	23.2
Not provided	6.7	24.3	18.3
Washington group - Budapest intitiative 2 short sets			
Seeing			
No difficulty	90.1	72.6	72.8
Some difficulty	6.7	23.6	24.4
Much difficulty	1.7	3.4	2.7
Unable	1.5	0.4	0.1
Hearing			
No difficulty	82.9	85.9	89.9
Some difficulty	12.1	11.9	8.5
Much difficulty	3.5	2.0	1.5
Unable	1.5	0.2	0.1
Washing and dressing			
No difficulty	96.3	93.6	94.4
Some difficulty	1.4	4.0	3.1
Much difficulty	1.0	1.0	1.1
Unable to do alone	1.3	1.4	1.4
Walking or climbing steps			
No difficulty	86.3	84.2	86.6
Some difficulty	6.4	8.8	7.2
Much difficulty	2.9	4.5	3.9
Unable to do alone	4.4	2.5	2.3
Remembering or concentrating			
No difficulty	71.6	-	-
Some difficulty	25.0	-	-
Much difficulty	3.4	-	-
Concentrating			
No difficulty	-	-	83.4
Some difficulty	-	-	11.7
Much difficulty	-	-	4.9
Remembering			
No difficulty	-	92.4	-
Difficulty	-	7.6	-
Communicating			
No difficulty	94.7	-	-
Some difficulty	4.1	-	-
Much difficulty	1.2	-	-
		-	-
Overall WG-SS or BI-M2 categorization			
No difficulty in any activity	55.5	46.6	46.3
Some difficulty but not much difficulty in any activity	30.2	39.6	38.0
At least one activity with much difficulty ou unable to do	14.3	13.8	15.7

Supplementary Table 1 (continued). Description of the samples studied (HSM and ESPS surveys). All figures are weighted percentages unless otherwise indicated.

	HSM Survey (N=23,348)	2012 ESPS Survey (N=15,315)	2014 ESPS Survey (N=17,593)
Activities of daily living (ADL)			
Feeding			
No difficulty	98.6	97.8	98.4
Some difficulty	0.4	1.3	0.8
Much difficulty	0.3	0.4	0.3
Unable to do alone	0.7	0.5	0.5
Toileting			
No difficulty	99.2	97.4	98.1
Some difficulty	0.2	1.7	1.0
Much difficulty	0.1	0.4	0.4
Unable to do alone	0.5	0.6	0.5
Dressing			
No difficulty	97.3	94.8	95.9
Some difficulty	1.2	3.6	2.7
Much difficulty	0.7	0.6	0.7
Unable to do alone	0.8	0.8	0.7
Bathing			
No difficulty	97.0	95.0	95.4
Some difficulty	0.9	2.7	2.3
Much difficulty	0.8	0.9	0.9
Unable to do alone	1.3	1.4	1.4
Transferring from bed or chair			
No difficulty	98.3	94.3	96.2
Some difficulty	0.7	4.4	2.6
Much difficulty	0.4	0.8	0.6
Unable to do alone	0.6	0.5	0.6
Walking			
No difficulty	91.1	88.8	89.9
Some difficulty	3.6	5.9	5.3
Much difficulty	1.9	3.3	3.0
Unable to do alone	3.4	2.0	1.8
Stineman ADL categorization, limitation:			
None	90.0	85.8	88.5
Mild	8.7	10.1	9.1
Moderate	0.2	0.8	0.3
Severe	0.6	2.3	1.2
Complete	0.5	1.0	0.9
Instrumental activities of daily living (IADL)			
Shopping			
No difficulty	93.6	90.7	91.2
Some difficulty	1.3	3.7	3.6
Much difficulty	1.2	1.4	1.4
Unable to do alone	3.9	4.2	3.8
Preparing meals			
No difficulty	97.0	93.2	94.8
Some difficulty	0.6	2.3	2.1
Much difficulty	0.6	0.7	0.5
Unable to do alone	1.8	3.8	2.6
Doing light housework			
No difficulty	94.1	91.1	91.4
Some difficulty	1.7	3.7	4.7
Much difficulty	1.6	1.5	1.5
Unable to do alone	2.6	3.7	2.8
Doing heavy housework			
No difficulty	92.3	79.1	81.8
Some difficulty	1.6	9.8	9.0
Much difficulty	1.8	3.7	3.2
Unable to do alone	4.3	7.4	6.0
Handling finances			
No difficulty	94.3	86.3	88.3
Some difficulty	1.4	6.4	5.9
Much difficulty	1.0	1.8	1.4
Unable to do alone	3.3	5.5	4.4
Using telephone			
No difficulty	98.8	96.9	97.4
Some difficulty	0.2	1.4	1.3
Much difficulty	0.2	0.6	0.4
Unable to do alone	0.8	1.1	0.9
Stineman IADL categorization, limitation:			
None	88.7	73.0	76.7
Mild	2.7	8.1	6.8
Moderate	5.0	12.6	10.5
Severe	2.8	4.7	4.5
Complete	0.8	1.6	1.5
GALI			
Not limited at all	71.7	69.6	70.7
Limited but not severely	17.4	20.9	20.3
Severely limited	10.9	9.5	9.0

Supplementary Table 2 Spearman's correlation coefficient matrix between individual items of BI-M2, ADL, IADL, and GALI. ESPS surveys, 2012 (above the diagonal) and 2014 (below the diagonal).

	BI-M2 items					ADL items						IADL items						
	Seeing	Hearing	Washing and dressing	Walking or climbing steps	Remembering or concentrating*	Feeding	Toileting	Dressing	Bathing	Transferring from bed or chair	Walking	Shopping	Preparing meals	Doing light housework	Doing heavy housework	Handling finances	Using telephone	GALI
Seeing	1.00	0.17	0.14	0.19	0.11	0.11	0.11	0.13	0.14	0.14	0.17	0.17	0.11	0.15	0.17	0.15	0.14	0.21
Hearing	0.20	1.00	0.19	0.24	0.11	0.13	0.15	0.18	0.18	0.18	0.22	0.18	0.19	0.19	0.22	0.17	0.19	0.21
Washing and dressing	0.18	0.22	1.00	0.50	0.21	0.43	0.62	0.90	0.87	0.65	0.53	0.58	0.47	0.57	0.48	0.33	0.39	0.39
Walking or climbing steps	0.24	0.27	0.54	1.00	0.19	0.25	0.37	0.44	0.47	0.45	0.84	0.56	0.35	0.51	0.58	0.34	0.30	0.52
Remembering or concentrating*	0.21	0.16	0.22	0.22	1.00	0.21	0.22	0.19	0.22	0.19	0.19	0.22	0.20	0.22	0.19	0.21	0.24	0.17
Feeding	0.12	0.15	0.47	0.30	0.18	1.00	0.56	0.46	0.47	0.44	0.27	0.34	0.39	0.35	0.25	0.25	0.40	0.19
Toileting	0.12	0.16	0.59	0.37	0.18	0.60	1.00	0.62	0.68	0.57	0.41	0.44	0.42	0.46	0.33	0.27	0.41	0.26
Dressing	0.16	0.20	0.87	0.48	0.22	0.50	0.66	1.00	0.71	0.65	0.48	0.52	0.44	0.51	0.43	0.29	0.37	0.35
Bathing	0.17	0.22	0.91	0.52	0.21	0.51	0.64	0.74	1.00	0.62	0.51	0.56	0.48	0.56	0.44	0.33	0.43	0.34
Transferring from bed or chair	0.17	0.18	0.68	0.49	0.21	0.48	0.63	0.71	0.64	1.00	0.46	0.49	0.40	0.47	0.43	0.28	0.34	0.34
Walking	0.21	0.26	0.57	0.87	0.21	0.34	0.41	0.51	0.56	0.52	1.00	0.58	0.38	0.53	0.55	0.34	0.31	0.47
Shopping	0.21	0.24	0.61	0.61	0.24	0.37	0.44	0.53	0.61	0.51	0.63	1.00	0.56	0.69	0.61	0.43	0.42	0.44
Preparing meals	0.15	0.21	0.51	0.42	0.21	0.44	0.47	0.49	0.52	0.45	0.45	0.60	1.00	0.63	0.47	0.40	0.45	0.31
Doing light housework	0.20	0.21	0.59	0.59	0.25	0.37	0.44	0.53	0.58	0.49	0.60	0.74	0.62	1.00	0.64	0.43	0.42	0.41
Doing heavy housework	0.24	0.25	0.50	0.62	0.25	0.27	0.31	0.43	0.47	0.40	0.58	0.64	0.48	0.68	1.00	0.43	0.32	0.55
Handling finances	0.20	0.24	0.36	0.39	0.24	0.27	0.29	0.32	0.37	0.30	0.37	0.45	0.42	0.44	0.46	1.00	0.38	0.30
Using telephone	0.15	0.23	0.40	0.33	0.20	0.47	0.44	0.40	0.43	0.37	0.35	0.45	0.50	0.43	0.33	0.40	1.00	0.24
GALI	0.24	0.23	0.39	0.53	0.26	0.21	0.25	0.35	0.36	0.33	0.49	0.46	0.32	0.46	0.56	0.32	0.24	1.00

* remembering in 2012 and concentrating in 2014

Supplementary Table 3 Factor pattern matrices obtained using principal component analysis with varimax (A) and promax (B) rotations for the 19 items of the WG-SS, ADL, IADL, and GALI disability indicators. HSM survey. Two components were extracted according to Horn parallel analysis and Velicer's MAP (see text). Loadings > 0.4 and 0.60, respectively, are highlighted in bold.

	A. Varimax rotation		B. Promax rotation	
	Factor 1	Factor 2	Factor 1	Factor 2
Seeing	0.32	**0.54**	0.48	**0.61**
Hearing	0.11	**0.65**	0.32	**0.65**
Washing and dressing	**0.90**	0.32	**0.96**	0.59
Walking or climbing steps	**0.87**	0.21	**0.89**	0.47
Remembering or concentrating	0.23	**0.76**	0.47	**0.80**
Communicating	0.31	**0.81**	0.55	**0.86**
Feeding	**0.82**	**0.40**	**0.91**	**0.64**
Toileting	**0.88**	0.34	**0.94**	**0.60**
Dressing	**0.89**	0.31	**0.94**	0.57
Bathing	**0.89**	0.34	**0.95**	**0.60**
Transferring from bed or chair	**0.88**	0.26	**0.92**	0.53
Walking	**0.88**	0.20	**0.90**	0.47
Shopping	**0.85**	0.39	**0.93**	**0.63**
Preparing meals	**0.82**	**0.46**	**0.93**	**0.69**
Doing light housework	**0.87**	0.36	**0.94**	**0.62**
Doing heavy housework	**0.85**	0.34	**0.91**	0.58
Handling finances	**0.68**	**0.58**	**0.83**	**0.76**
Using telephone	**0.63**	**0.64**	**0.80**	**0.80**
GALI	**0.75**	0.30	**0.81**	0.52

Supplementary Table 4 Rasch analyses of dimensionality and differential item functioning (DIF*) for the BI-M2, ADL, and IADL items (recoded as binary variables, limited vs non-limited). ESPS 2012 survey.

BI-M2 items	Fit with the one-dimensional Rasch model			Differential item functioning	
Overall fit p-value=0.0001, Person separation index=0.34	location	Chi square	P-value	Uniform DIF	Non-Uniform DIF
Seeing	-1.05	4.52	0.10	–	–
Hearing	-1.39	3.47	0.18	–	–
Washing and dressing	0.08	13.80	0.001	education, occupation	–
Walking or climbing steps	1.50	8.67	0.01	gender, age, education, occupation, income, couple	–
Remembering	0.86	4.70	0.10	–	–

ADL items	Fit with the one-dimensional Rasch model			Differential item functioning	
Overall fit p-value<0.0000001, Person separation index=0.82	location	Chi square	P-value	Uniform DIF	Non-Uniform DIF
Feeding	1.61	8.18	0.04	–	–
Toileting	1.52	7.97	0.05	–	–
Dressing	-0.44	20.19	0.0002	–	–
Bathing	-0.15	21.41	0.00009	–	–
Transferring from bed or chair	-0.59	7.39	0.06	–	–
Walking	-1.95	31.01	0.000001	–	–

IADL items	Fit with the one-dimensional Rasch model			Differential item functioning	
Overall fit p-value<0.0000001, Person separation index=0.81	location	Chi square	P-value	Uniform DIF	Non-Uniform DIF
Shopping	0.14	14.80	0.002	gender, couple	–
Preparing meals	0.45	3.24	0.36	gender, couple	–
Doing light housework	0.23	24.01	0.00003	gender, age	–
Doing heavy housework	-2.13	5.71	0.13	–	–
Handling finances	-1.02	18.33	0.0004	occupation	–
Using telephone	2.32	2.87	0.41	–	–

* A difference of 0.1 logit was considered to indicate meaningful DIF (see text)

Supplementary Table 5. Rasch analyses of dimensionality and differential item functioning (DIF*) for the BI-M2, ADL and IADL items (recoded as binary variables, limited vs non-limited). ESPS 2014 Survey.

BI-M2 items	Fit with the one-dimensional Rasch model			Differential item functioning	
Overall fit p-value=0.04, Person separation index=0.45	location	Chi square	P-value	Uniform DIF	Non-Uniform DIF
Seeing	-0.92	2.21	0.53	–	–
Hearing	-1.25	2.92	0.40	gender	–
Washing and dressing	1.85	6.70	0.08	education	–
Walking or climbing steps	0.37	10.61	0.01	–	–
Concentrating	-0.06	3.50	0.32	–	–

ADL items	Fit with the one-dimensional Rasch model			Differential item functioning	
Overall fit p-value<0.0000001, Person separation index=0.83	location	Chi square	P-value	Uniform DIF	Non-Uniform DIF
Feeding	2.04	8.93	0.03	–	–
Toileting	2.01	6.05	0.11	–	–
Dressing	-0.49	16.78	0.0008	–	–
Bathing	-0.59	18.49	0.0004	–	–
Transferring from bed or chair	-0.30	8.63	0.03	–	–
Walking	-2.68	21.99	0.00007	–	–

IADL items	Fit with the one-dimensional Rasch model			Differential item functioning	
Overall fit p-value<0.0000001, Person separation index=0.84	location	Chi square	P-value	Uniform DIF	Non-Uniform DIF
Shopping	-0.04	20.42	0.0004	gender, couple	–
Preparing meals	0.77	5.38	0.25	gender	–
Doing light housework	0.04	23.19	0.0001	–	–
Doing heavy housework	-2.17	4.92	0.30	occupation	–
Handling finances	-0.97	25.51	0.00004	gender, occupation	–
Using telephone	2.36	3.19	0.53	–	–

* A difference of 0.1 logit was considered to indicate meaningful DIF (see text)

Supplementary Fig. 1.

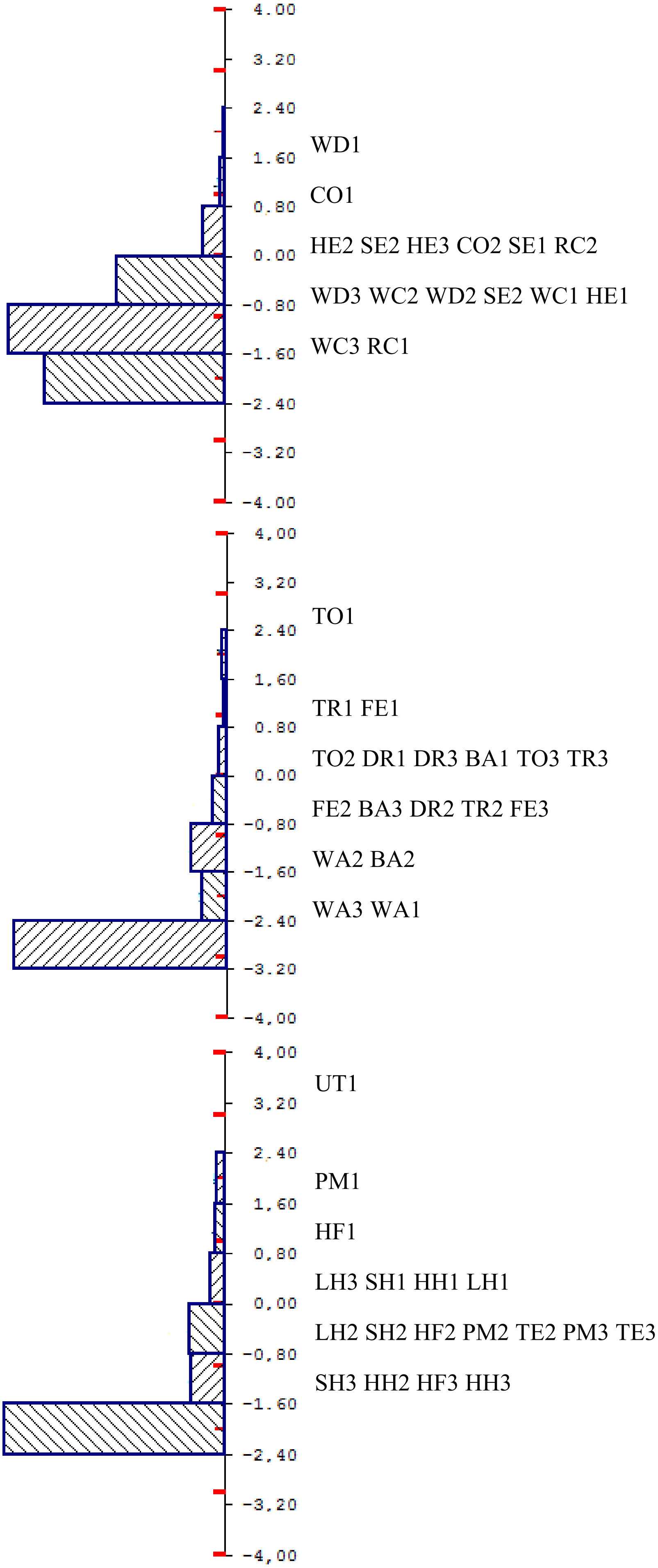

Supplementary Fig. 2.

Subject estimates Item estimates

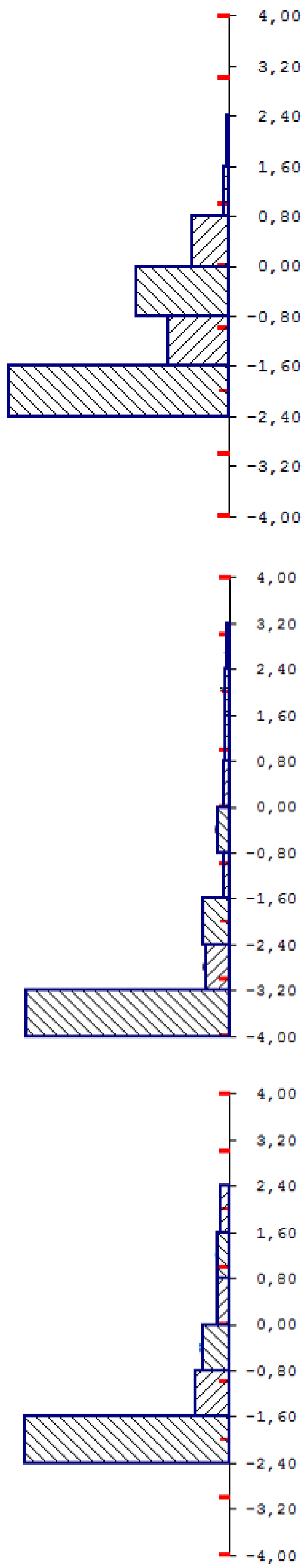

Supplementary Fig. 3.

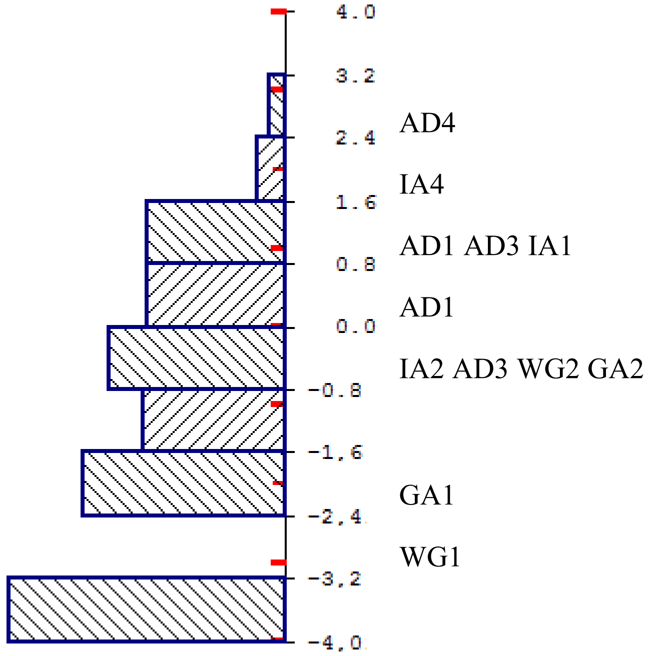

Supplement: Supplementary file 1 — Additional file 1: Supplementary Table 1. Description of the studied samples (HSM and ESPS surveys). Supplementary Table 2. Spearman’s correlation coefficient matrix between individual items of BI-M2, ADL, IADL, and GALI. ESPS surveys, 2012 and 2014. Supplementary Table 3. Factor pattern matrices obtained using principal component analysis with varimax and promax rotations for the 19 items of the WG-SS, ADL, IADL, and GALI disability indicators. HSM survey. Supplementary Table 4. Rasch analyses of dimensionality and differential item functioning for the BI-M2, ADL, and IADL items (recoded as binary variables, limited vs non-limited). ESPS 2012 survey. Supplementary Table 5. Rasch analyses of dimensionality and differential item functioning for the BI-M2, ADL and IADL items (recoded as binary variables, limited vs non-limited). ESPS 2014 Survey. Supplementary Fig. 1. Subject-item maps of the WG-SS, ADL, and IADL items (four-category responses or three thresholds, 1: some difficulty, 2: much difficulty, 3: unable to do alone; two-category responses and one threshold: “some difficulty or more”). HSM survey. On the left of the diagram are the subjects, and on the right are the thresholds of each item (point on the continuum where the response category “some difficulty or more” is most likely to be chosen by a subject with the corresponding level of disability). Less disabled subjects are near the bottom of the diagram, and most disabled subjects are near the top. Abbreviations SE: Seeing, HE: Hearing, WD: Washing and dressing, WC: Walking or climbing steps, RC: Remembering or concentrating, CO: Communicating. FE: Feeding, TO: Toileting, DR: Dressing, BA: Bathing, TR: Transferring from bed or chair, WA: Walking. SH: Shopping, PM: Preparing meals, LH: Doing light housework, HH: Doing heavy housework, HF: Handling finances, UT: Using telephone. Supplementary Fig. 2. Subject-item maps of the WG-SS, ADL, and IADL items (three-category responses or two thresholds, 1: some dif [file 13690_2023_1164_MOESM1_ESM.pdf]
